# Supplementary material for: Zwitterionic Polymer Coating Enabled Chronic Dopamine Sensing and Electrophysiology Recording in Free‐Moving Mice
Source: Adv Healthc Mater. 2026 Sep 13;15(37):e71688. doi: 10.1002/adhm.71688 (PMC13650806; doi:10.1002/adhm.71688)
Supplement: Supplementary file 1 [file ADHM-15-0-s001.docx]

**Supporting Information**

**Zwitterionic polymer coating enabled chronic dopamine sensing and electrophysiology recording in free-moving mice**

Bingchen Wu^1,2^, Cort Thompson^1^, Thomas Deakin^3^, Yan Xu^3^, Colleen McClung^4^, Xinyan Tracy Cui^1,2,5, #^

1. Department of Bioengineering, University of Pittsburgh. Pittsburgh, PA,15213, USA.
2. Center for the Neural Basis of Cognition, Pittsburgh, PA, 15213, USA.
3. Department of Anesthesiology and Perioperative Medicine, Pittsburgh, PA, 15213, USA.
4. Department of Psychiatry, University of Pittsburgh. Pittsburgh, PA,15213, USA.
5. McGowan Institute for Regenerative Medicine, Pittsburgh, PA 15219, USA

\# Corresponding Author: xic11@pitt.edu

Figure S1. a), Comparison of EIS spectrum of PSB coated PEDOT/CNT microelectrodes using subcutaneously inserted fresh reference electrodes vs. brain-implanted reference electrodes over 4-weeks of implantation. b), Comparison of EIS spectrum of PSB-coated PEDOT/CNT MEAs and non-coated PEDOT/CNT MEAs using subcutaneously inserted fresh reference electrodes over 4-weeks of implantation.

Figure S2. Spatial and temporal patterns of SNR for both groups. (a), The PSB-coated group showed a much more stable SNR over 4 weeks. (b), The non-coated group showed more variable SNR and even loss of sortable single units on some channels after 2 weeks.

Figure S3: SEM comparison of PSB-coated and non-coated explants at 4 weeks.

(a) PSB coated (b) Non-coated
